# Primary Afferent Nociceptor as a Target for the Relief of Pain

**DOI:** 10.1155/2012/348043

**Published:** 2012-03-01

**Authors:** Carlos A. Parada, Claudia H. Tambeli, Paul G. Green, Brian E. Cairns

**Affiliations:** ^1^State University of Campinas, 13083-862 Campinas, SP, Brazil; ^2^Department of Oral and Maxillofacial Surgery, University of California San Francisco, San Francisco, CA 94143-0440, USA; ^3^Faculty of Pharmaceutical Sciences, University of British Columbia, Vancouver, BC, Canada V6T 1Z3

 Primary afferent nociceptors (A-delta and C fibres) are generally the first structures to be involved in the perception of pain. These specialized primary afferent sensory neurons have also been described as important to the development of inflammatory and neuropathic pain. Primary afferent nociceptors are particularly interesting as a target for the development of new drugs to control pain given the fact that they are located outside the central nervous system and separated from it by the blood brain barrier. Although significant advances have been made to better understand the molecular mechanisms involved in activation of primary afferent nociceptors, important aspects of this process remain unclear. In this special issue are papers that address some of these aspects.

Dr. R. Hulse and colleagues discuss the importance of galanin and the galanin receptor 2 on primary afferent neurons as a potential target to control neuropathic pain. As described by the authors, despite the dramatic increase of galanin expression in the peripheral nervous system following nerve injury, few studies have investigated the role that this neuropeptide plays in modulating nociception at the level of the primary afferent nociceptor. It is important to highlight that the most widely used medicines to control neuropathic pain include anticonvulsants (antiseizure medications) and antidepressants and that these agents are usually used for central nervous system disorders. As a result, these therapies for neuropathic pain are associated with significant central nervous system side effects, such as drowsiness, dizziness, and cognitive deficits. Therefore, the finding of increased expression of galanin in primary afferent nociceptors could emerge as an interesting peripheral target to control neuropathic pain that might be devoid of problematic central nervous system side effects.

Dr. V. Chaban summarizes the role that estrogen plays in visceral nociception at the level of primary sensory neurons. This review paper reports that the incidence of persistent, episodic, or chronic “functional” visceral pain associated with disorders such as irritable bowel syndrome, fibromyalgia, painful bladder syndrome, chronic pelvic pain, and others is much more prevalent in women than in men. Based on this observation, the author hypothesizes that viscerovisceral cross-sensitization is mediated by estrogen modulation of the response of dorsal root ganglion neurons to adenosine triphosphate (ATP). This estrogen-mediated modulation of visceral afferent nociceptors may also explain the observed clinical and animal sex-related differences in visceral hypersensitivity.

Dr. Y. Tousignant-Laflamme and Dr. S. Marchand hypothesize that aging, independent of the hormonal status, changes pain responses in young postmenopausal women. They tested this hypothesis by examining pain responses to different experimental nociceptive stimuli in a group of young postmenopausal women and compared the results to nonmenopausal women. They undertook these experiments while the nonmenopausal women were menstruating, since sex hormone levels are at their lowest during menses. Blood sampling enabled them to assure that both groups had comparable sex hormones levels for progesterone and estrogen, the main female sex hormones. The authors concluded that age seems to be the main factor influencing changes in tonic pain perception in the group of midlife postmenopausal women. They also suggested that a reduction of the peak pain intensity in the postmenopausal women was probably due to a reduction of function in myelinated A*δ* fibers that occurs naturally with age.

Dr. Y.-H. Jin and colleagues hypothesize that glutamate is released in peripheral tissues following activation of the TPRV1 receptors. This increase in peripheral glutamate then acts on glutamate receptors on primary afferent nociceptor terminals to activate nociceptors. They tested this hypothesis by measuring c-Fos expression in the spinal cord following injection of capsaicin alone (to activate TRPV1 receptors) and capsaicin with various glutamate receptor antagonists. Neuronal c-Fos expression was significantly increased by capsaicin, and this effect was antagonized when capsaicin was coadministered with glutamate receptor antagonists. Based on the effectiveness of the different glutamate antagonists used, the authors conclude that glutamate receptors are present on peripheral terminals of primary afferent nociceptors and that activation of these ionotropic glutamate receptors mediate hyperalgesia produced by activation of TRPV1.

Dr. K. E. Miller and colleagues describe findings of an increase in the enzyme glutaminase in primary afferent sensory neurons during inflammation. They hypothesize that elevated glutaminase action in primary afferent nociceptor cell bodies could produce increased glutamate synthesis in the peripheral and spinal terminal endings of these neurons. This increase in glutaminase activity may contribute to the mechanisms that underlie central sensitization and may also provide a potential target for novel therapies for the treatment of chronic pain.

The paper by M. Gautam and colleagues examines the underlying mechanism of pain in a model of long-lasting mechanical hyperalgesia that employs repeated injections of a low-pH solution into the gastrocnemius muscle at 5-day intervals. One unanswered question about this model is whether the hyperalgesia is mediated by a long-term alteration in the expression and/or response properties of acid-sensing ion channels (ASICs) on muscle nociceptors. Their paper combined whole cell patch clamp and behavioral testing to determine whether long-term changes in the properties of peripheral ASICs contribute to the mechanical hyperalgesia seen in this model. The authors conclude that ASICs are not involved in long-term maintenance of hyperalgesia in this model.

*Carlos A. Parada* *Carlos A. Parada* *Claudia H. Tambeli* *Claudia H. Tambeli* *Paul G. Green* *Paul G. Green* *Brian E. Cairns* *Brian E. Cairns*

